# Biomembrane models and drug-biomembrane interaction studies: Involvement in drug design and development

**DOI:** 10.4103/0975-7406.76461

**Published:** 2011

**Authors:** R. Pignatello, T. Musumeci, L. Basile, C. Carbone, G. Puglisi

**Affiliations:** Department of Drug Sciences, University of Catania, viale A. Doria, 6 - 95125 Catania, Italy

**Keywords:** Biomembrane models, cell membrane, DSC, Immobilized Artificial Membrane (IAM) chromatography

## Abstract

Contact with many different biological membranes goes along the destiny of a drug after its systemic administration. From the circulating macrophage cells to the vessel endothelium, to more complex absorption barriers, the interaction of a biomolecule with these membranes largely affects its rate and time of biodistribution in the body and at the target sites. Therefore, investigating the phenomena occurring on the cell membranes, as well as their different interaction with drugs in the physiological or pathological conditions, is important to exploit the molecular basis of many diseases and to identify new potential therapeutic strategies. Of course, the complexity of the structure and functions of biological and cell membranes, has pushed researchers toward the proposition and validation of simpler two- and three-dimensional membrane models, whose utility and drawbacks will be discussed. This review also describes the analytical methods used to look at the interactions among bioactive compounds with biological membrane models, with a particular accent on the calorimetric techniques. These studies can be considered as a powerful tool for medicinal chemistry and pharmaceutical technology, in the steps of designing new drugs and optimizing the activity and safety profile of compounds already used in the therapy.

## Drug-biomembrane Interaction Studies in Biomedical and Pharmaceutical Research

The human body possesses a variety of biomembranes, whose functions range from protecting tissues and cells from foreign molecules, and to select the cellular penetration of compounds with a biochemical or physiological role.

After its administration, a drug molecule rapidly meets many of these biomembranes, from the circulating macrophage cells to the vessel endothelium, to the more complex blood-brain or blood-retinal barriers. Consequently, drug distribution in the body is largely affected in terms of time and concentration.

Besides a structural role, biomembranes play other essential functions: control the passage of selected compounds, thus maintaining the biochemical integrity of cytosol; communication, allowing the exchange of information between the extra- and intracellular environments, and the physical interaction with the extracellular phase; biochemical-active surface, due to the amount of associated enzymes, receptors, ion channels, signaling molecules, and supramolecular structures that have a role in cellular homeostasis, metabolism, growth, and even death. Therefore, pathological alterations in the structure or functions of cell membranes and other biomembranes are often caught up in the etiology of many diseases.

Studying the molecular events occurring on cell membranes, as well as the multiplicity of interactions with bioactive compounds, in either physiological or pathological situations, is therefore of paramount importance, to enlarge our knowledge of many diseases and to identify further potential therapeutic targets.

Interactions of drugs and biological compounds with biomembranes are complex phenomena from the chemical or physicochemical point of view. They can represent a final stage, in case the biomembrane embodies a barrier to drug passage or is the site of action for the drug (e.g., at the level of the membrane receptors). In many cases, however, drug-membrane interaction represents only a preliminary step to the biological (or toxic) activity, as it can affect the rate of penetration and partitioning of the biomolecule in the cytoplasm, to reach a specific target cell organelle or system. In other words, both partitioning into and binding with cell membranes deserve to be accurately studied and characterized, for the old as well as the novel bioactive compounds.[1]

It becomes evident that a drug-membrane interaction can be considered either as a partitioning phenomenon or a binding pathway. To generalize a situation that is instead often complex and multivalent, when the membrane acts as a barrier to the drug penetration into cells, that is, when the pharmacokinetic aspects are considered, the partitioning phenomena are more important. Conversely, when the cell membrane represents the site of action for the drug (pharmacodynamic), the drug binding processes deserve to be explored. These aspects become greatly relevant when the right model biomembranes are to be designed for *in vitro* studies.

The forces underlying both kinds of interactions are the same, that is, polar and hydrophobic chemical interactions.[1] On this basis, although there is a great complexity of biochemical phenomena occurring in living cells, it is relatively simple to stress on such simplification, and design an efficient experimental model, suitable for investigating or even predicting the possible drug-membrane interactions.

The results of such interactions can be reciprocal, in the sense that the biomolecule can alter the structure and function of the membrane, for instance change its permeability, charge potential, fluidity, and so on; but, on the other hand, the structure and properties of the drug can also be affected by its interaction with the membrane components, in terms of stereochemistry, molecular conformation, time of onset, and duration of the biological activity, for instance.[2,3]

A passive, relatively easy diffusion through the lipid domains of the biomembranes has been considered since a long time, as being the main process regulating the permeability of drugs across membranes and the whole cell internalization process. The pharmacokinetics and pharmacodynamic patterns of drugs have been shaped according to such a statement. More recently, however, the role of membrane transporters has been highlighted. Large superfamilies of transporter proteins have been found in every living cell.[4] In a recent review, Dobson *et al*. have discussed the role of such proteins in the cell uptake of drugs, and of the modeling strategies that can be used to forecast drug pharmacokinetic features.[5]

Nevertheless, it is still valid to deem that interaction with the cell surface is a prerequisite for drug activity, even when such an interaction is not followed by its internalization into the cytoplasm. The so-called ‘non-specific’ interaction of drugs with the biomembrane components involves a contact with the phospholipid (PL) structures, which can affect the highly organized lipid compartment and cause changes in the membrane protein conformation and work.[6]

For analogous reasons, drug-biomembrane interactions can be the basis for adverse or toxic side effects,[7] such as phospholipidosis.[4,8] The presence of a foreign compound can damage membrane integrity: amphiphilic compounds, in particular, like many classes of drugs are, can exert a detergent-like activity on membranes, causing their disruption and dysfunction.[9,10]

As natural cell membranes present a great complexity of structure, cross-connections and functionality, artificial model membrane systems, under intensive development, help the scientists to understand the effects of membrane lipids in drug transport and uptake into cells, drug activity, and even toxicity. In respect to living cell membranes, these models can be used under conditions that will not allow the cells to remain live and biochemically integral.

This review will illustrate some of the analytical techniques applied in the study of interactions among bioactive compounds, with biological membrane models, with particular emphasis on calorimetric methods. This kind of data can be a powerful tool for medicinal chemistry and pharmaceutical technology, to design and optimize the activity and tolerability profiles of new drugs. Moreover, ‘old’ compounds also, whose clinical usefulness has been limited by their physicochemical properties, and thereby by pharmacokinetic problems, can be drag to a second life by optimizing precise details in their chemical structure.

## The Structure and Functions of Cell Membranes

**Figure 1:**
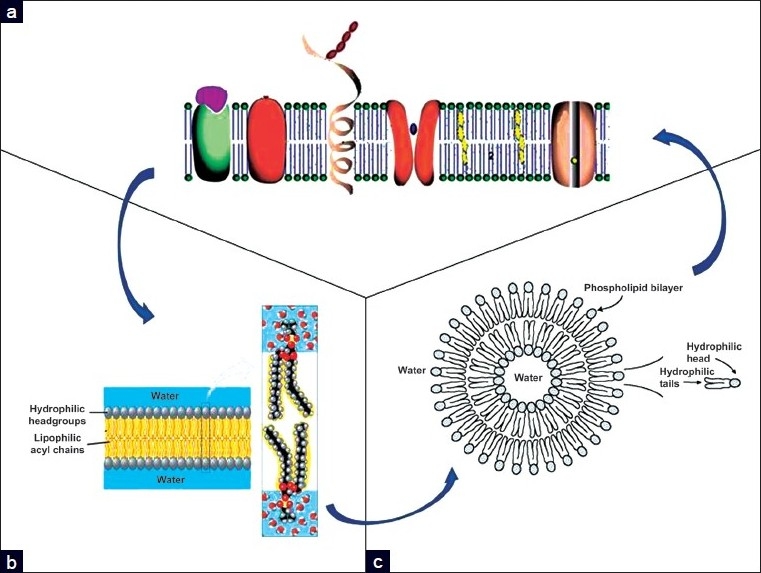
The relations are shown among cell membranes (a), phospholipid bilayers (b), and liposomal vesicles (c)

Membranes of eukaryotic cells are made of three major components: lipids, proteins, and sugars. All membranes have a common general structure [Figure 1a], with structural or functional proteins (e.g., enzymes, receptors, channels, etc.) embedded within the two-layered sheets of lipid molecules. The lipid and protein molecules are held together mainly by non-covalent interactions, and alterations of their dynamics and strength are often associated with diseases. Sugars are attached by covalent bonds to some of the lipids and proteins. They are found on one side of the membrane only, for example, on the outer surface of the plasma membrane.

Biological membranes contain three major kinds of lipids: PL, glycolipids, and cholesterol. PL is generally formed by glycerol linked to two fatty acids, a phosphate group, and a basic head group; the fatty acid chains usually contain 14 to 24 carbon atoms. One chain can be unsaturated, containing from one to four *cis* double bonds. The three major glycerol-based phospholipids contain choline, serine, or ethanolamine attached to the phosphate; another type of phospholipid contains sphingosine instead of glycerol, such as sphingomyelin. About 40% of the lipids in eukaryotic cells are phosphatidylcholines (= lecithins), which are zwitterionic in a pH range from 4 to 10.

Thus, they carry one negative and one positive charge in the physiological pH range.

A common attribute of membrane lipids is their amphipathic nature. Both PL and glycolipids have a hydrophilic head and two hydrophobic tails: in an aqueous medium, these molecules spontaneously associate to form bilayers, with their hydrophobic tails sandwiched between the hydrophilic heads [Figure 1b].

The PL bilayers can be further simplified as a fluid phase containing three specific domains: a nonpolar hydrocarbon core, an interfacial region containing the uncharged PL acyl ester groups, showing an intermediate polarity, and the highly polar membrane surface that contains the charged PL head groups that is exposed to the aqueous exterior. These bilayers tend to close on themselves to form sealed compartments called liposomes, to eliminate the edges where the tails would be in contact with the water [Figure 1c].

A small drug molecule or an ion that migrates from the surface of a PL bilayer to the inner domains encounters a remarkable decrease in polar solvation and dielectric constant. Indeed, the low polarity of the hydrocarbon chain domain hinders the penetration of the charged or polar species, and most proteins that span a bilayer membrane have a sequence of nonpolar amino acids that match the thickness of the hydrocarbon region.

**Figure 2:**
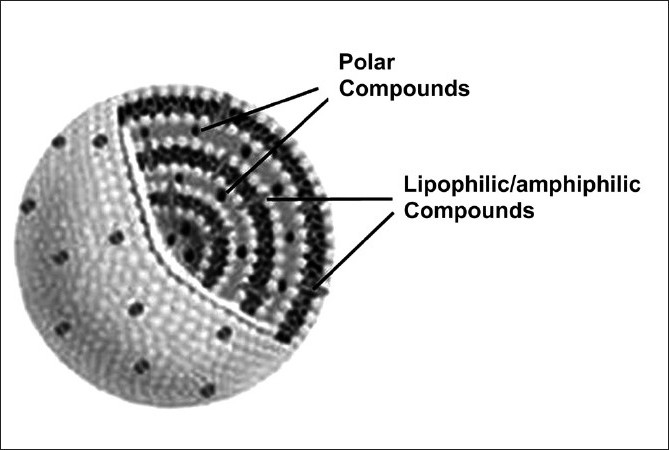
Schematic structure of a multilamellar liposome, showing the possible location of the host compounds: lipid soluble or amphiphilic compounds (gray ellipsoids) allocate completely or in part among the PL acyl chains and hydrophilic compounds (black spots) are retained in the aqueous spaces between the bilayers

Cholesterol contains a four-ring steroid structure together with a short hydrocarbon side-chain and a hydroxyl group. Cholesterol is found in some mammalian membranes, but not in most bacterial membranes, nor in plant membranes. As an amphipathic molecule, it can be incorporated into PL bilayers, but cannot form a bilayer on its own[Figure 2].

The cell membrane is currently not considered as the homogeneous and static bilayer, as described in the classical Singer and Nicolson’s ‘fluid mosaic model’, but as a heterogeneous medium with a complex and dynamic lipid organization at a nanoscale level.[11] Simons and Ikonen proposed the concept of ‘functional rafts’ to describe the lateral domains rich in saturated lipids and cholesterol, dispersed within a phase rich in unsaturated lipids.[12] These structures are endowed with membrane proteins and can further be involved in chemical interactions with other functional proteins and sugars. Moreover, their size and structure may dynamically change under specific signals or stimuli (or also in some disease states) underlying the multiplicity of cellular responses.[13]

Both the lipids and proteins are able to move within their own monolayer in the membrane. Lipid membranes can undergo transitions from a rigid to a liquid crystal lamellar state, where both the lateral order of the lipid molecules and the order of the lipid hydrocarbon chains change. In biological membranes this occurs at temperatures slightly below the body temperature. Both states form two-dimensional membranes, but their physical properties may be quite different. The main factors that determine the fluidity of cell membranes, apart from the temperature, are the length and the degree of unsaturation in the fatty acid tails of PL, the characteristics of their head groups, and the concentration of cholesterol in the membrane. This aspect retains a relevant role in the development and validation of biomembrane models.

In contrast with the rapid lateral diffusion, lipid molecules rarely move from the monolayer that they are in to the opposite one, and often the lipid composition of the two layers is quite different. The transfer of a PL molecule from one layer to the other - known as transverse diffusion or *flip-flop* - is rare, because the polar, hydrophilic head would have to penetrate the non-polar, hydrophobic hydrocarbon core of the bilayer.

## The Elaboration of Artificial Biomembrane Models

Cell and biological membranes essentially consist of a lipid environment where liposoluble compounds can dissolve and pass through. Therefore, among the physicochemical features that characterize a small bioactive molecule in terms of interaction with living cells, solubility and partition coefficients are the most important.[14]

As the interaction with membranes can represent only the first step of a cascade of chemical and physical processes, such as relations with protein receptors, enzymes or nucleotides, it is actually the balance between the hydrophilic and lipophilic characters of a molecule, that is, its *amphiphilicity* that determines a successful interaction with biomembranes.

Many studies have shown that the simple parameter of lipophilicity cannot result in an overall improvement of cellular uptake of a drug or its passage through the barriers. The composite structure of cell membranes, as previously described, involves that drugs must possess an amphiphilic character to be able to modulate their movements inside both the lipid domains and polar spaces of the membranes.[15] The complex phenomena linked to partitioning into and binding of the drug to the cell membranes or barriers are better related to the so-called ‘anisotropic lipophilicity’.[1] It not only derives from the hydrophobicity of drugs, but also from its ability to make polar and ionic bonds with the membranes.

The traditional way to express the lipophilicity / amphiphilicity of a compound has been the determination of its partition coefficient (logP) between two immiscible liquid phases, typically, a water or a buffered aqueous solution and an organic solvent.[16–18] The cross-evaluation of the logP value in a four-solvent series allowed to draw a more complete lipophilicity/hydrophilicity profile of the test compounds.[19] All these 2-D systems share the relative simplicity of use and remain of great value when, for instance, a chemically related series of analogs must be compared or quantitative structure-activity relationship (QSAR) studies on large sets of compounds must be developed.

However, when the aim is the prediction of the interaction with biomembranes, solvent–solvent partition experiments suffer the absence of a tridimensional scaffold, similar to that involved in side-interactions between living cell membrane components and a drug. Hydrogen bonds or van der Waals interactions are, for instance, difficult to reproduce or simulate in an isotropic 2-D liquid system. Moreover, the partitioning of chargeable or charged compounds is almost impossible to measure and reproduce.[20]

In the last few years, many alternative models have thus been developed, in which a 3-D structure is built to allow as many interactions as possible with the test compound. The well-defined composition and structure of these complex assemblies can allow to better reproduce the phenomena that occur *in vivo* during drug transport, distribution, biological activity, and even resistance. Brilliant examples of experimental comparison between *n*-octanol-water logP data and 3-D membrane models can be found in the fundamental work of Seydel and Wiese (2002).[6] Another excellent review by Peetla *et al*.[7] has recently examined the available biomembrane models and discussed their utility in studying the interaction with different classes of drugs, as well as in predicting their efficacy or toxicity.

Artificial membrane models allow the carrying out of particular studies in experimental conditions, such as temperature or osmolarity, at which the living cells cannot be driven without damaging their functionality. In these models some cell membrane properties are obviously lost, such as the presence of functionalized membrane proteins, receptors, endocytosis, and other active processes; thereby, the biophysical interactions of drugs with artificial membranes may not exactly reproduce all aspects of the biological environment. However, a good reproducibility of results and correlation with *in vitro* as also *in vivo* pharmacological or toxicological behavior has been observed in many investigations.

Generally speaking, three main kinds of lipid membrane models have been identified: monolayers, vesicle-forming bilayers (liposomes), and supported bilayers. An interrogation of the Pubmed database on the articles published in the last 10 years using such biomembrane models gave approximately 500 hits, many of which made use of calorimetric techniques [Table 1].

**Table 1:** Literature overview of recent investigations using DSC and other techniques to study the interaction of drugs with various biomembrane models

Biomembrane model	Analytical tool(s)	Host drug/Compound	Refs.
Phospholipid vesicles (SUV, LUV, MLV) and Micelles	DSC, LB, BAM, Fluo	Melatonin	De Lima *et al*., J. Pineal Res. 2010;49,169
			
	DSC	E-3,5,4’-trimethoxystilbene/β-CyD	Sarpietro *et al*., Int J Pharm. 2010;388,144.
	DSC	Labaditin	Barbosa *et al*., Amino Acids 2010
	DSC, BAM, PM-IRRAS	Plasticins	Joanne el al., Biochemistry 2009;48,9372
	DSC, Fluo, DLS	BC5 (N-pentadecylpiperidin-4-amine)	Luciani *et al*., Mol. Biosyst. 2009;5,356
	DSC, XRD	NSAIDs	Lucio *et al*., Langmuir. 2008;24,4132
	ITC	Cathelicidin antimicrobial peptides	Andrushchenko *et al*., BBA 2008 1778;1004
	DSC	Xenobiotics	Zepik *et al*, Crit Rev Toxicol. 2008;38,1
	DSC	Thymopentin prodrugs	Pignatello & Pecora, Pharmazie 2007;62,663
	DSC	(-)-Epicatechin conjugates	Lazaro *et al*., J Agric Food Chem. 2007;55,2901
	DSC, XRD, FF	Sulfadiazin	Oszlánczi *et al*., Biophys Chem. 2007;125,334
	DSC	N-oxides of tertiary amines	Kleszczynska *et al*., 1 Naturforsch C. 2005;60,567
	DSC	Gemcitabine	Castelli *et al*., J Colloid Interface Sci. 2005;285,110
	DSC	β-interferon derivatives	Larios *et al*., Biophys Chem. 2004;111/123
	DSC	Tocopherols and phenolic compounds	Gutiérrez *et al*, Life Sci. 2003;72,2337
	DSC	rBPI(21)	Domingues *et al*., PLoS One. 2009;4,e8385.
	DSC, Fluo	Acyclovir and squalenoyl-acyclovtr	Sarpietro etal., Int J Pharm. 2009;382,73
	DSC	Plantaricin 149 synthetic peptides	Lopes *et al*., BBA 2009;1788,2252
	DSC	D-cycloserine	Musumeci *et al*., J Liposome Res. 2008;18,211
	DSC, TEM	Bilirubin-IXalpha enantiomers	Ceccacci *et al*., Bioorg Chem. 2008;36,252
	DSC	Chlorpromazine	Zhang *et al*., Biochem. Biophys ResCommun. 2008;373,202
	DSC	Gemcitabine and prodrugs	Castelli *et al*., J Colloid Interface Sci. 2007;3,1643
	DSC	Lipoamino acids (LAA)	Pignatello *et al*., CurrDrug Deliv. 2007;4(109
	DSC	resveratrol and derivatives	Sarpietro *et al*., J Agric Food Chem. 2007;55,3720
	DSC	β-Sitosterol/β-CyD	Castelli *et al*., J Agric Food Chem. 2006;54,10228
	DSC	Oxicams	Lúcio *et al*., Med Chem. 2006,2,447
	DSC	Docetaxel loaded-PLA/PLGA	Musumeci *et al*., Int J Pharm. 2006;325,172
	DSC	Herbicides	Librando *et al*., Environ Sci Technol. 2006,40,2462
	DSC	Idebenone amphiphilic prodrugs	Pignatello *et al*., J Colloid Interface Sci. 2006,299,626
	DSC	Tranylcypromine amphiphilic conjugates	Pignatello *et al*., Int J Pharm. 2006,310,53
	Fluo	Tocopherols and tocotrienols	Sonnen *et al*., J Am Chem Soc.2005,127,15575
	DSC	Drug release from polymer conjugates	Castelli *et al*., Drug Deliv. 2005,12,357
	HPLC	Biphenyl derivative	Ceccacci *et al*., J Am Chem Soc. 2005,127,13762
	DSC	Arginine-based cationic surfactants	Castillo *et al*., Langmuir.2004;20,3379
	UV-Vis, Fluo	Quinolones antibiotics	Neves *et al*., Biophys Chem. 2005,113,123
	DSC	Hepatitis G virus envelope protein peptides	Larios *et al*., Langmuir 2004;20,11149
	NMR, ROESY	Ditryptophan, diphenylalanine	Bombelli *et al*., J Am Chem Soc. 2004,126,13354
	DSC	Micronized nimesulide	Castelli *et al*., Eur J Pharm Sci. 2003;19,237
	DSC, mol. modeling	Ofloxacin	Fresta *et al*., Bioorg Med Chem. 2002;10,3871
	DSC	Papaverine in CyDs	Ventura *et al*., J Drug Target.2001;9,379
	Fluo	(-t-)-Totarol	Mateo *et al*., BBA 2000;1509,167
	Spectroscopic and other techniques	HAV-VP3 peptides	Sospedra *et al*., Biopolymers 2000,54,477
	CFM, DSC	Chitosan microspheres loaded with moxifloxacin	Ventura *et al*, Eur J Pharm Biopharm. 2008;68,235
	DSC	Inulin-based hydrogel	Castelli *et al*., Eur J Pharm Sci.2008;2,76
Monolayers	LB	Chromium(III) complexes	Sella *et al*., BBA, 2010
	LB	Local anesthetics, alcohols	Frangopol *et al*, Colloids Surf B Biointerfaces 2001;22:3.
	LB	Acyclovir and prodrugs	Sarpietro *et al*., Int J Pharm. 2010
	LB	Frutalin lectin	Nobre *et al*., BBA 2010;1798;1547
	LB	Plantaricin 149 peptide analog	Lopes *et al*., BBA 2009;1788,2252
	LB	Gemcitabine and squalene prodrug	Castelli *et al*., J Colloid Interface Sci.2007;3,1643
	LB	Gemcitabine prodrugs	Castelli *et al*., J Colloid Interface Sci. 2007; 313,363
	DLS	cationic liposome-DNA complexes	Uchiyama *et al*., Anal Sci.2004,20,1537
Supported bilayers	AFM	Melatonin	De Lima *et al*., J. Pineal Res. 2010;49,169
	Real-time AFM	Triton X-100	Morandatand El Kirat, Langmuir 2006;22,5786
	HCM, Fluo	Antimicrobial peptides	Davis *et al*., J. Pept Sci. 2009;15,511
	AFM	Oritavancin	Domenech *et al*., BBA 20091788,1832
	AFM	Protegrin-1	Lame *et al*., J. Phys. Chem. B. 2006;110,21282
Phospholipidcoated columns (Immobilized artificial membranes)	HPLC	(-)-Epicatechin conjugates	Lazaro *et al* J Agric Food Chem. 2007;55,2901
	HPLC	–	Zhang *et al*., J. Sep. Sci., 2010, in press
	HPLC	Various model drugs	Zhang *et al*., Talanta. 2005;67,1023
	HPLC	Flavonoids	Ollila *et al*., Arch Biochem Biophys. 2002;399,103
	Molecular chormatography	Statins	Sarr t al., J Chromatogr B Analyt Technol Biomed Life Sci. 2008; 868,20
	HPLC	Various model drugs	Barbato *et al*., Eur J Pharm Sci. 2004;22,261
Immobilized phospholipid capillary electrophoresis	HPLC	NSAIDs	Mei *et al*., Talanta 2008;75,104

Abbreviations: AFM: atomic force microscopy; BAM: Brewster angle microscopy; CD: circular dichroism; CFM: Confocal fluorescence microscopy; CyD: cyclodextrin; DLS: dynamic (quasi-elastic) light scattering; DSC: differential scanning calorimetry; FF: freeze-fracture; Fluo: fluorescence spectroscopy methods; HCM: hyperspectral confocal microscopy; ITC: Isothermal titration calorimetry; LB: Langmuir-Blodgett monolayer films; LUV: large unilamellar vesicles; MLV: multilamellar liposomes; NMR: nuclear magnetic resonance spectroscopy; PM-IRRAS: Polarization modulation infrared reflection absorption spectroscopy; ROESY: rotational nuclear overhauser effect spectroscopy; SEM: scanning electron microscopy; SUV: small unilamellar liposomes; TEM: transmission electron microscopy; TR-QELS: time-resolved quasi-elastic laser scattering; XRD: X-ray diffraction methods.

As amphiphilic molecules, PL form monomolecular insoluble films (called *Langmuir monolayers*) on the surface of a liquid subphase. Such films are excellent model systems to study membrane biophysics, because a biological membrane can be considered as two weakly coupled monolayers.[21,22] They can be useful models for analyzing the structure and the mixing interactions of drugs with PL monolayers, at the air-water interface.[15,23–27] Moreover, their extemporary method of production allows to use lipids with the necessary physicochemical properties.

To study the interaction of drugs with PL, a lipid / drug mixture is spread over water or an aqueous buffered phase to form a monolayer in a Langmuir trough. The monolayer is then compressed, and the surface pressure-area isotherms are measured and compared to the one of pure lipids.[22] Two main thermodynamic parameters, namely surface pressure and temperature, can be easily controlled in this application.

Apart from being valid drug nanocarriers, *liposomes* have become interesting models of biomembranes to study the interaction with drugs and to make partitioning experiments.[27–30]

Liposomes are self-assembling systems usually composed of PL molecules. Bilayers made of native PL, extracted from different kinds of cell membranes, have also been described.[2] Hydration of these lipids with an aqueous medium spontaneously produce concentric multilamellar vesicles (MLV). The mechanical handling of the latter by sonication or extrusion through membrane filters, yields unilamellar liposomes of different size (SUV, LUV). Partitioning experiments can be performed using either MLV or SUV suspensions, in which a drug or another foreign compound can be incorporated during vesicle production, or, as revealed in the review of Castelli *et al*. in this issue, allowed to diffuse inside preformed, void vesicles.

**Figure 3:**
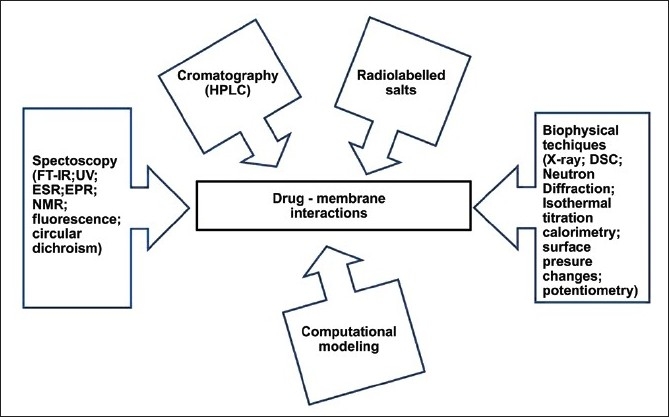
Outline of the main analytical techniques applied for studying the interaction between drug or drug carriers and biomembrane models

**Figure 4:**
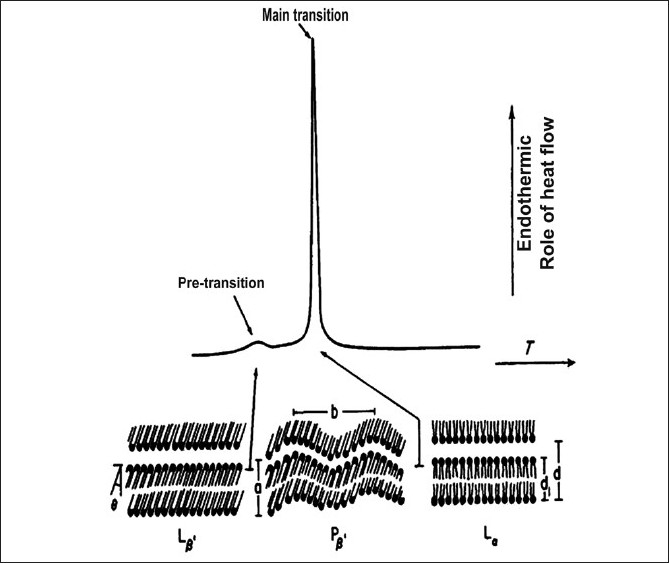
Typical DSC curve of phospholipid bilayers undergoing gel-to-liquid crystal phase transition under controlled heating

The amphiphilic nature of PL produces liposomes with an ordered bilayer arrangement, with the acyl chains aligned to form a lipid domain and the polar head groups (e.g., choline, serine) facing the external or interlamellar aqueous spaces [Figure 3]. According to its chemical nature, an extraneous compound can thus find room within the acyl chains (as hydrophobic and aromatic molecules do), in the aqueous spaces (for hydrophilic or polar compounds), or even between them, if an amphiphilic compound is hosted [Figure 4]. Artificial bilayers show many physical features very close or comparable to natural membranes, such as, thickness, electrical resistance, interfacial tension, and so on.[31]

In spite of specific problems linked to the solubility of the host compound or to the compatibility or degradation issues, liposomes generally represent a valid means to study the partition of biologically related substances. The main advantage of liposome models is their anisotropicity, which allows the occurrence of tridimensional phenomena between the host molecule and the PL bilayers. Thus, steric hindrance and spatial-related troubles, which can affect the *in vivo* behavior of a drug, can be definitely observed and exploited. Lúcio *et al*.,[32] for example, has shown that as the evaluation of the antioxidant properties, correlated with the activity of non-steroidal anti-inflammatory drugs, receives an additional value using a 3-D liposome model, the effects of the drugs on the lipoperoxidation of membrane components can be analyzed as a function of drug-membrane interactions.

In other words, while *partitioning* is the only phenomenon that can be reproduced in solvent–solvent models, with 3-D anisotropic models also, the physicochemical and chemical processes can be generated, to simulate at least in part the *binding* phenomena that occur in living systems.[1]

A further, very intriguing issue is that of the electrical charge of membranes and / or host molecules. Although on one hand the differences in the partition profiles of neutral and protonated species, often noticed with the octanol-water system, are basically reduced with liposomes[6] so that experiments can be carried out within a large range of physiologically-related pH values; on the other hand, cell membranes show a net surface charge, which can influence electrostatic interactions with drugs and biomolecules. Such types of interactions might not be predictable with the bidimensional solvent / solvent partition experiments, but they can be measured or predicted using 3-D systems made of suitably charged PL or lipids.[33]

*Supported lipid bilayers* can be formed on atomically smooth solid supports, such as silicon, by the Langmuir-Blodgett / Langmuir-Schäfer (LB / LS) technique,[34] by the vesicle fusion (VF) technique[35] or by a mixed Langmuir-Blodgett / vesicle fusion (LB / VF) technique.[36] These systems are also valid models for reproducing the thermodynamics of cell membranes, even when different liquid lipid phases coexist.[37] On contact and interaction with the test drugs, changes in morphology, structure, and chemistry of these supported bilayers can be monitored by coupled analytical techniques, such as Fourier transform infrared spectroscopy (FTIR), scanning electron microscopy (SEM), transmission electron microscopy (TEM), X-ray diffractometry, atomic force microscopy (AFM), and so on.[38]

The use of these various 3-D membrane models has contributed to the unraveling of many limits associated with isotropic solvent-solvent systems, such as the impact of an electrical charge or the stereochemistry and spatial orientation of bulky parts of the molecules, on their interactions with the membranes. However, all these models still present severe limitations when a direct transfer of the experimental information to living biological systems or when a prediction of the *in vivo* behavior of a drug is endeavored.

## Analytical Techniques Available to Study Drug-Membrane Model Interactions

This paragraph will give some quick references to the principal methodologies that have been explored or proposed in the last few years, for this special type of study. More detailed information on how each technique works and can be used, may be found in some of the cited references.

Many analytical methods have been investigated with the aim of determining the affinity of compounds for biomembranes [Figure 3]. Chromatography (HPLC), spectroscopy (FT-IR, UV, ESR, EPR, NMR, fluorescence depolarization, circular dichroism, SIMS), and other biophysical techniques (X-ray and neutron diffraction methods, differential scanning calorimetry (DSC), isothermal titration calorimetry, surface pressure changes, potentiometry, etc.), often in combination with each other. Of late in-depth investigations have been allowed, to find out the effects of drug molecules on the structural and functional aspects of cell membranes. Using radiolabeled salts (displacement of ^45^Ca ^++^) is also a relatively simple tool to carry out drug-membrane affinity experiments. In recent years, different computational modeling approaches have also been directed to these studies.[39]

Some of these applications have been recently reviewed by Seddon *et al*.[40] and more information can be found in the textbook of Seydel and Wiese.[6]

We basically agree with other authors in their consideration that to characterize the interactions of drugs with biomembrane models well, from a qualitative and quantitative point of view, no single technique can give all the required data, however, the association of complementary experiments could provide utmost benefits.

Among the chromatographic methods set up for this particular application, Immobilized Artificial Membrane (IAM) chromatography allows to efficiently simulate liposome / water partitioning and cell membrane permeation.[41–44] IAM stationary phases are solid-phase systems, where a phospholipid monolayer is covalently bonded to a propylaminosilica support material. With IAM columns, simple aqueous mobile phases (e.g., PBS) can be used, without addition of organic modifiers. With respect to liposomal vesicles, the hydrophobic domain is half large in the IAM surface and the PL is more ordered and less mobile. Solute partitioning between the eluent and stationary phases seems to be the mean retention mechanism in IAM retention.[41] However, polar interactions are often involved, depending on the structural properties of the analytes. Thus, protonated basic compounds are stronger retained, because of their interaction with the phosphate anionic groups of the stationary phase.[45] The potential of IAM chromatography is to predict passive transport through various biological barriers, as well as to estimate drug pharmacokinetic and pharmacodynamic properties, which has been recently reviewed.[46–48]

As for liposomes, the results of IAM chromatography give mixed information on passive diffusion (permeation) of drugs and drug-membrane interactions (binding), although the contribution of electrostatic forces and hydrogen bonds has been reported to be weaker in IAM chromatography than in liposome partitioning[49,50] Correlation with *n*-octanol-water lipophilicity[51] and the quantitative structure-retention relationship with IAM chromatography, using classical physicochemical molecular descriptors, has been elucidated.[52] However, it would seem that the results obtained with these techniques can be efficaciously compared to the interaction profile with biomembranes only for strictly structure-related classes of molecules.[53]

In more recent years, other types of biomimetic stationary phases have been developed, like α_1_-acid glycoprotein or albumin derivatized surfaces, widening the applications of biochromatography as a reliable tool to study drug interaction with cells and biosystems.[54]

A further advance in this field is represented by biopartitioning chromatography (BPC), a technique in which a chromatographic method is combined with biomembrane-mimetic structures like PL vesicles or monolayers, polymer micelles, microemulsions, niosomes, and so on. In the last few years BPC has become a high-throughput screening platform to study drug-membrane interaction and permeability and their correlation with the biological effects.[55]

Also, the capillary electrophoresis technique (EC) has been tailored to create a valid model for drug-membrane interaction studies.[56] EC experiments have been used to characterize the size, surface properties, encapsulation volumes, and the electrophoretic mobility of colloidal lipid vesicles and of lipoprotein particles. Interactions between biologically-related compounds and lipid vesicles that serve as pseudostationary phases, or as coated stationary phases, in electrokinetic chromatography, can be used to investigate the biophysical nature of drug-membrane model interactions.

Among the spectroscopic techniques, NMR analysis, and especially the new, more sensitive techniques, such as Nuclear Overhauser Effect (NOE) or transfer NOE, can add more detailed information about the interactions occurring at a molecular level between a drug and lipid and PL molecules.[57] These experiments, in some cases, allow to identify the molecular specificities responsible for the interaction with and / or permeation of drugs through the membrane bilayers.[58]

Solid-state NMR (SS-NMR) is also a valid technique to explore, in depth, the effects of the host compounds on the package, and inter-connections among biomembrane components. Thus, ^2^H, ^13^C, ^31^P, and ^15^N SS-NMR, or a combination of the various techniques, allow to determinate the degree and level of interaction of model compounds with the PL bilayers, as well as the effects on the morphology and physicochemical property of the resulting membranes. In many cases, it becomes possible to discriminate between the effect exerted on the polar surface or on the hydrophobic alkyl chain domain of the bilayers, monitoring the interaction with foreign molecules, for example, as a function of PL composition and charge, temperature, and pH.[59,60]

A fluorescence study, using the fluorescent probe 1,6-diphenyl-1, 3, 5-hexatriene (DPH), has been applied to test the effects of NSAIDs on different membrane systems, including liposomes, mouse macrophages, a human leukemia monocyte cell line, granulocytes, and mononuclear cells. The tested NSAIDs were able to efficiently quench the probe located in the membrane hydrocarbon region and to enhance the membrane fluidity, proving their interaction with the membrane lipids. Authors suggested that the induced changes in lipid dynamics could affect the activity of inflammatory enzymes or could be related to the local side effects of NSAIDs on the stomach mucosa.[61]

Fluorescence assays have been also used to follow the binding and transport of fatty acids (FA) in model and biological membranes. Partitioning of FA between membranes and the aqueous buffer, insertion of the FA acyl chain into the hydrophobic core of the phospholipid bilayer, and the presence of the FA carboxyl group at the outer leaflet of the membrane have been studied using different probes.[62]

A luminescence assay, which is based on the energy transfer of a permeant to liposomal terbium (III) has also been used to examine drug permeation in the membrane bilayers.[63] The experimental results on model acidic molecules led to the important conclusion that depending on single variables like membrane composition and rigidity or electrostatic interactions, and on the geometry of the model system, lipid bilayer permeation may positively, negatively or not correlate with the bilayer affinity of the tested molecule. Small angle X-ray diffraction and small angle neutron scattering techniques have been utilized to identify the position of drugs in model and native multi-bilayer vesicles.[2] An improved potentiometric evaluation of the lipid membrane - water partition coefficient of ionizable drugs has been recently described, in which the data analysis was corrected on the basis of Coulomb electrostatic phenomena.[64] Voltammetric methods have been also applied to analyze the ionic transfer kinetics of ionizable drugs across the lipid-modified liquid-liquid interfaces.[65] This technology promises interesting developments in the field of high-throughput assessment of the ADMET properties of drugs.

## Calorimetric Techniques

Calorimetric approaches are among the most used techniques to study drug-biomembrane interactions. DSC is a diffuse technique for such studies.[6,66–68] In DSC experiments, the thermotropic changes, eventually occurring in a liposome sample, in the presence of a drug molecule, are registered and quantified. The concept statement of DSC is that PL vesicles undergo a reversible phase transition, under the effect of increasing temperature, from a ‘gel’ state, in which the acyl chains are orderly packed within the bilayers, to a ‘liquid crystal’ state, associated with an increase of spatial disorder in the bilayers [Figure 4]. Such a transition is joined with heat uptake, therefore, it is an endothermic process. A detailed description of the technique can be found in many reviews (e.g.,[68–71]) and is illustrated in other articles published in the present issue.

Extensive DSC studies from some of us and from Castelli and coworkers have been focused on the effects on DPPC or DMPC (dimyristoylphosphatidylcholine) uni- and multi-lamellar liposomes or different series of analogs.[26,72–78]

The DSC analysis of the interactions of xenobiotics, such as drugs, with liposomal models may also correlate with their biological and toxicological behavior.[1,79–84] Also the toxicological pattern of drugs can be marked out;[85] for instance, Jelokhani-Niaraki *et al*. used isothermal titration calorimetry to assess the lytic effects on model PL membranes, for the conformational changes occurring in gramicidin S analogs.[86]

When the fatty acid chains of PL are in their (solid) crystalline state, they are in an extended conformation and in *all trans* configuration. They form a regular crystal lattice, stabilized by low energy forces and electrostatic interactions between the polar head groups. During the phase transition to liquid crystal, the melting of the acyl chains causes a passage to the gauche configuration, leading to an increase of chain mobility.

**Figure 5:**
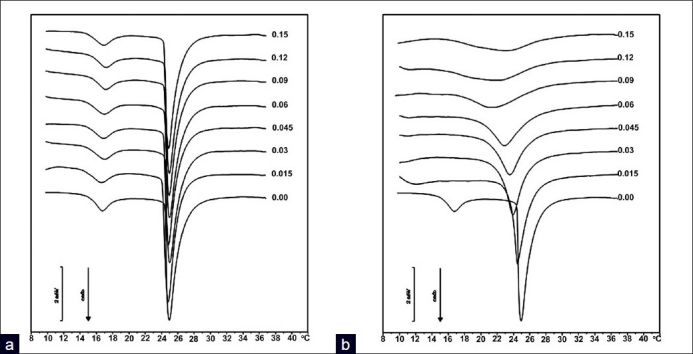
DSC curves of MLV liposomes made of pure DMPC or containing increasing molar fractions (Xo = 0-0.15) of a model host compound. (a), a compound exhibiting a reduced interaction with the PL bilayers (e.g., a polar molecule) induced limited thermotropic effects; (b) strong deformation of the DSC curves of PL bilayers under the influence of a lipophilic compound (F. Castelli, *personal data, with permission*)

Addition of an extraneous compound, based on its chemical nature and thus its location within the bilayers, is able to induce a defined change in the thermotropic parameters of the liposomes, with respect to pure PL vesicles. For instance, a reduction in the temperature of the main phase transition (Tm) can be essentially ascribed to an interference with the polar (choline) head groups of the PLs; similarly, variations in calorimetric enthalpy changes (ΔH) associated to this phase transition rather denote the degree of interaction with PL acyl chains. In Figure 5 a typical example is given, where increasing the molar fractions of a host compound can either induce a progressive lowering of the Tm of pure dimyristoylphosphatidylcholine (DMPC) MLV liposomes or not, along with a reduction in the associated ΔH changes, according to the degree of interaction with the bilayers.

Other parameters can help to understand the occurring interaction, for instance, the shape of the endothermic transition peak, expressed as the width at half the height of the peak itself (T_½_), which gives a clear indication of the cooperativity of the system in the presence of a foreign compound, that is, the uniformity of distribution of the latter within the bilayers, from the surface through the core of liposomes.[87.^88^] Also the transition interval, that is, the temperature range between the onset and the end of the phase transition curve, is indicative of the homogeneity of the host compound-liposome system and its cooperativity. A detailed explanation of the fundamentals of this technique can be found in this same issue, in the review of Chiu and Prenner.

## Conclusions

As this review belongs to an issue focused on special applications of calorimetric techniques, and DSC in particular, we have pointed out some examples derived from our study and studies of other authors, confirming the utility of such experimental approaches.

Analysis of the interactions that can occur between a drug or a biologically active compound, and biomembranes are becoming a settled part of the design, discovery, and characterization of new drugs, and progressively at an earlier stage of development. Many different analytical techniques have been applied or developed on purpose to perform these kinds of studies. Using artificial membranes as simplified models for cell membranes has given a strong input to the understanding of the complex set of interactions that a biomolecule can develop toward biological membranes, and often also vice versa.

However, the addition of an external compound to a PL bilayer or monolayer can induce physicochemical changes on its own, which will confuse the interpretation of the experimental data. Consequently, it is a lot more evident that reliable and constructive data may be acquired only on the basis of two elements: (i) the availability of new and more sophisticated models for cell and biological membranes, although simple enough to be used and reproduced; and (ii) the concurrent application of different analytical techniques, whose specific contribution will help to give an overall consciousness of these interactions.
